# Exploring Strategies to Mitigate the Adverse Health Impacts of Air Pollution on Children in India: A Qualitative Study

**DOI:** 10.7759/cureus.64630

**Published:** 2024-07-16

**Authors:** Deepti Chhabra, Katayoun Jahangiri, Sanaz Sohrabizadeh, Zohreh Ghomian, Abbas Shahsavani

**Affiliations:** 1 Department of Health in Disasters and Emergencies, School of Public Health and Safety, Shahid Beheshti University of Medical Sciences, Tehran, IRN; 2 Department of Environmental Health Engineering, School of Public Health and Safety, Shahid Beheshti University of Medical Sciences, Tehran, IRN

**Keywords:** air pollution, strategies, children, health, india

## Abstract

Background: Air pollution poses a significant threat to global public health, contributing to high rates of mortality and morbidity. India, home to the world's largest population of children, is particularly affected. This study aims to identify effective strategies to mitigate the adverse health impacts of air pollution on this vulnerable group.

Material and methods: The study utilized directed content analysis using a deductive approach and purposeful sampling to carry out in-depth interviews with researchers, academicians, paediatricians, public health experts, and climate change experts from different organizations in India. In total, 17 interviews were conducted over two months in March and April 2024 until data saturation was reached.

Results: A total of 29 subcategories were extracted. The main sub-categories include strategies for reducing indoor emissions and multisectoral emission reduction, strategies to reduce exposure at home, schools and transit, strategies for public awareness, effective communication, health sector communication and awareness, and raising awareness by frontline workers and educational institutions, strategies for capacity building of health sector and frontline stakeholders, strategies for building research and knowledge translation, strategies for vertical and horizontal collaboration, strategies for child-centric policies, school closure policies, fiscal policies, comprehensive policymaking, sectoral policymaking, advocacy in policymaking, strategies for monitoring, and strategies for mother and child health.

Conclusions: The results of this study indicate that mitigating the adverse health impacts of pollution for children would entail a multi-pronged approach encompassing effective communication and education strategies and awareness raising of important stakeholders such as health professionals, community, grassroots-level workers, parents, teachers and children. Such strategies could be useful to trigger the desired change in behaviour of all concerned. Also, there is a need for collaboration and partnership between various stakeholders and ministries as policy-making bodies. There is a need to build on the research and strengthen the monitoring and surveillance.

## Introduction

Air pollution is one of the biggest global health threats. World Health Organisation (WHO) General Director, Dr. Tedros referred to air pollution as a “silent public health emergency” and “the new tobacco” during the first WHO Global Conference on Air Pollution and Health held in 2018 [1]. The severity of this emergency is brought about by the fact that household air pollution (HAP) alone led to about 3.2 million people deaths in 2020, mostly in low- and middle-income countries (LMICs) [2]. Premature deaths due to air pollution occur mainly on account of heart disease, stroke, chronic obstructive pulmonary diseases, and lung cancer in adults and acute respiratory infections in children [3]. Further, diseases and deaths due to air pollution not only have an economic impact but also a societal impact due to absences from productive work and school and subsequent loss of man-days [1]. Though the evidence in favor of this has mostly emerged from research in high-income countries, according to the Global Burden Of Disease (GBD) study, the largest burden of disease lies in LMICs [4].

Within this gamut of LMICs, the impact of pollution in Asian cities is even more profound with cities in India occupying the top 50 positions amongst the most polluted cities. Even within this group, the case of India is the most alarming as 13 of the 20 most polluted cities in the world are in India [4]. Air pollution poses a significant public health threat in India, which constitutes 17% of the world’s population [4]. India contributed 18.1% of the global population but had 26.2% of the global air pollution disability-associated life years (DALYs) in 2017 [5].

Among the most adversely affected cities in Asia, is the national capital of India, New Delhi. Despite being the country's capital and receiving adequate resources and governmental attention, Delhi was ranked as the most polluted capital city in the world in 2019 with an annual average concentration of particulate matter 2.5 (PM2.5) of 440 µg/m^3^. It houses approximately 26 million residents [6]. Although there is significant exposure and associated negative health outcomes, research in India is behind because of the absence of comprehensive air pollution exposure assessment and its subsequent integration with health data to investigate health effects [4]. It is important to note that some mega capital cities such as London and Tokyo have controlled their air pollution levels with appropriate legislation and stricter control, whereas capital cities such as Delhi in the developing world are still grappling with this issue [7]. 

Air pollution affects the health of all but its impact on children is more damaging [8]. Ambient air pollution (AAP) contributed to 4.2 million premature deaths in 2019, with 154,000 deaths of children under five years of age, while HAP was responsible for 3.2 million premature deaths, with over 237,000 deaths of children under five [9]. Children are particularly vulnerable to air pollution on account of their developing respiratory, nervous, and immune systems. Also, their inhalation rate is twice that of adults which, along with oral breathing, makes them more susceptible to particulate matter deposition in their lower respiratory tract [8]. 

The current interventions by the Government of India to mitigate pollution can largely be said to be under the framework of the National Clean Air Programme (NCAP) aimed at reducing pollution levels in a few cities across India with an urban focus, thus overlooking over 60% of the significant child population that resides in rural areas. It has largely ignored the health component of the public at large in general and that of children's health in particular [10]. Further, the impacts on children are expected to be compounded by global climate change, highlighting the urgent need for policymakers to promptly prioritize and address the mitigation of primary and secondary sources of air pollution [10].

Children in the age group of 0-14 years constitute 30.76% of the total population in India as per census 2011 [11]. Still, there is inadequate policy focus to protect their health against air pollution in India [10]. Adequately managing this cohort is vital for the reduction of disease burden and long-term impact as children are the prospective working population and their ill health would endanger their future. So, investment in their health is an investment for the future. The sheer number of man-hours lost on account of long-term health impacts such as morbidity and mortality caused by pollution for this cohort in the long run can be very significant. So far, the policies targeting air pollution in India have focussed on the aspect of emission reduction and have been generically targeting the whole population. Children have been treated as a subset of the larger population ignoring their vulnerabilities and susceptibilities. It is pertinent that the air pollution policies and interventions need to be customized to adequately protect children and adolescents specifically and not just as “little adults” [12].

The objective of this study is to explore the applicability of strategies derived from a prior study conducted by the authors [13]. The prior study was conducted by means of document analysis to derive strategies to protect the population’s health in the world [13]. In this study, the authors applied those categories for children’s health in India using directed content analysis using a deductive approach, a method beneficial for testing concepts, categories, and theories in a new context.

## Materials and methods

Study design and setting

This was a qualitative study applying a directed content analysis method with a deductive approach for extracting codes and subcategories. A deductive content analysis approach is used to validate an existing theory [14]. In the current study, the results obtained from document analysis conducted by the authors in their previous study [13] were used to test this theory in the context of children’s health in India. The eight categories derived by means of document analysis conducted by the authors [13] were applied in the current study. These categories include strategies for emission reduction, strategies for exposure reduction, educational and communication strategies, strategies for research, strategies for capacity building, strategies for policymaking, implementation and enforcement strategies, collaboration and coordination strategies, and strategies for evaluation.

Delhi was chosen as the setting as it is one of the most polluted cities in the world. The study was approved by the Shahid Beheshti University of Medical Sciences, Tehran, Iran (approval number: IR.SBMU.PHNS.REC.1402.047). Participants gave informed consent and confidentiality of personal information and the freedom to withdraw from the study were guaranteed. Participants were assured that the collected information shall be used solely for research purposes

Participants

Purposeful sampling was used to identify and select participants with the experience and knowledge keeping in mind the objective of the research. Further, their willingness and availability to participate in the research and communicate their experiences and opinions in an articulate manner were also considered [15]. The participants were researchers, academicians, pediatricians, public health experts, and climate change experts, chosen based on educational background, occupation, and area of expertise from different government and multilateral organizations located in India. Criterion-I was used to identify participants based on inclusion criteria. and snowball strategy was used to reach out to more participants [15]. A total of 25 participants were approached out of which 17 participated in the study. Most participants allowed recording of their voices except one participant. Hence handwritten notes were taken while interviewing him. The research environment was mainly the workplace of the participants, i.e. the premises of the institute, ministry, hospital, and multilateral agency. The participants were chosen from diverse backgrounds to have a holistic view of the possible strategies.

Inclusion criteria were people who had executive and scientific experience and were working in the areas of climate change, environment, air pollution, and health. Exclusion criteria were people who did not speak English or Hindi and who did not consent to participate.

Data collection

The participants were asked questions pertaining to the said categories. This was done to ensure that any new opinions could be used to complete the codes and provide new strategies in the context of children’s health in India. Data was collected through semi-structured interviews. The researcher referred to the potential participants, explained the research objectives, and scheduled an interview if participants agreed to take part in the study. The interview guide consisted of open-ended questions based on eight extracted strategies in the context of children’s health in India. The follow-up questions were asked based on the responses from the participants. The main questions that the participants were asked are included in the Appendices.

Interviews were collected over a period of two months, March and April, 2024. The duration of the interviews ranged from 20 minutes to 70 minutes. Data saturation was reached when the final two interviews didn’t yield any new codes or themes. To have a holistic picture, diverse kinds of participants with diverse backgrounds with experience in the field of air pollution, climate change, and health were chosen.

Data analysis

Data analysis was performed through a deductive approach using the categorization matrix [14]. The process of data collection and data analysis was conducted concurrently. All interviews were recorded on voice memos on a laptop and transcribed on Microsoft Word software (Microsoft Corporation, Redmond, Washington, United States). The first author listened to the interviews, transcribed texts, and read them several times to make sense of the whole content. This was followed by the identification of meaning units which were then condensed and labelled as a code. These codes were then compared based on their similarities and differences, and the similar codes were placed in related categories and labeled with subcategories.

Trustworthiness

Guba and Lincoln's strategies were used to ensure trustworthiness [16]. To ensure credibility, participants from diverse backgrounds were chosen. Member and peer checks were done to ensure credibility and dependency. The confirmability and transferability of the data were ensured by providing details of the study. Site triangulation by choosing participants working at diverse places was chosen to ensure confirmability.

## Results

A total of 17 participants (five female and 12 male) in the age group of 31-69 years were interviewed, working in environment health (n=2), climate change and health (n=4), air pollution and health (n=3), public health (n=3), and respiratory medicine (n=5), with work experience in the range of 6-42 years. Their demographic information is presented in Table 1.

**Table 1:** Demographic characteristics of the participants (N=17)

Participant (P)	Gender	Age	Education	Expertise	Work Experience (Years)
P1	Female	46	Doctor of Medicine (Pediatrics)	Pediatric Pulmonology	19
P2	Male	47	Doctor of Philosophy (Environmental Engineering)	Air Quality, Climate Change & Health	17
P3	Male	59	Doctor of Philosophy (Environmental Engineering)	Environmental Management, Air Quality Research	33
P4	Male	69	Doctor of Medicine (Medicine)	Pulmonary Medicine	42
P5	Male	65	Doctor of Medicine (Pediatrics)	Pediatric Pulmonology	32
P6	Male	39	Doctor of Medicine (Community Medicine)	Public Health, Climate Change & Air Pollution	16
P7	Male	39	Master of Public Health	Climate Change & Health	13
P8	Male	67	Doctor of Philosophy (Faculty of Engineering)	Air Quality Modelling	35
P9	Male	37	Master of Technology (Environmental Engineering)	Air Pollution Control	15
P10	Female	36	Master of Public Health	Climate Change & Health	7+
P11	Male	46	Master of Sciences	Environmental Health	23
P12	Female	55	Doctor of Philosophy (Social Medicine)	Public Health	22
P13	Male	59	Doctor of Philosophy (Public Health & Epidemiology)	Public Health	33
P14	Female	43	Doctor of Medicine (Pediatrics)	Pediatric Pulmonology	17
P15	Male	53	Doctor of Medicine (Pediatrics)	Pediatric Pulmonology	25
P16	Male	33	Doctor of Medicine (Community Medicine)	Public Health, Climate Change	6
P17	Female	31	Master of Public Policy	Climate Change Mitigation	8

In the present study, a total of 910 codes were extracted during primary coding which were combined based on similarities into sub-subcategories resulting in eventually 29 subcategories (Table 2).

**Table 2:** Selected codes, sub-subcategories, and sub-categories of the strategies for mitigating adverse health impacts on children in India ASHA: accredited social health activists

Category	Selected Codes	Sub-subcategories	Sub-categories
Strategies for emission reduction	Reduce biomass use at homes	Clean fuel for home	Strategies to reduce indoor emissions
	Increase piped gas coverage		
	Clean techniques for cooking	Clean technology for home	
	Use clean chulha (hearth)		
	Flue-gas desulphurization (FGD) power plants	Reduce emissions by power sector	Strategies for multi-sectoral emission reduction
	Reduce coal use		
	Prevent garbage burning	Reduce emissions by waste management	
	Manage waste		
	Clean industry	Reduce emissions by industrial sector	
	Shift industry out		
	Stop parali (agricultural stubble) burning	Reduce emissions by agricultural sector	
	Sustainable agriculture		
	Dust addressing interventions	Reduce emissions by construction sector	
	Monitor construction activities		
	Better transport such as electric vehicles (EVs)	Reduce emissions by transport sector	
	Sustainable road transport planning		
	Health sector to use sustainable practices	Reduce emissions by health sector	
	Health sector to reduce emissions		
Strategies for exposure reduction	Reduce travel time to school	Reduce travel time	Strategies for exposure reduction during transit
	Reduce distance from schools		
	School timings in low traffic times		
	Travel to school in closed vehicles	Travel in cleaner vehicles	
	School buses be closed systems		
	Use filters in buses		
	High Efficiency Particulate Air (HEPA) filters in house	Improved ventilation	Strategies for exposure reduction at home
	Improve ventilation in urban slums		
	Keep children away from cooking area	Better practices	
	Stop smoking indoors		
	Stop idling of school buses	Address transport around schools	Strategies for exposure reduction at schools
	No vehicles zone around schools		
	Plan more green belts within schools	Green initiatives	
	Make school facilities greener		
	Use smart boards for schools	Innovative solutions	
	Water guns for school playgrounds		
	Shift the schools to low-pollution areas	Siting of schools	
	Remove industries around schools		
	Ventilation systems in schools	Design of schools	
	Air filtration unit for classrooms		
Strategies for education and communication	Educate community	Raise community awareness	Strategies for public awareness
	Messaging on ventilation		
	More awareness in rural areas		
	Raise awareness to know “Clean air as a right”		
	Health talks in schools	Awareness and education of children	
	Educate children through curriculum		
	Develop educational programmes		
	Enhance children’s participation in green drive		
	Education of parents	Educate families	
	Awareness of parents		
	Educate caregivers		
	Children to wear masks	Notify children	
	Limit activities of children during high pollution days		
	Health communication needs to be precise, specific, and consistent	Effective messaging	Strategies for effective communication
	Use people from same socioeconomic background for messaging		
	Develop awareness tools in local language	Awareness tools	
	Develop action-oriented awareness tools		
	Sensitize the health sector	Raise awareness of health sector	Strategies for health sector communication and awareness
	Reach practicing health professionals through national chapters		
	Re-imagining curriculum in undergraduate courses in medical colleges		
	Health professionals to create awareness	Messaging by health sector	
	Doctors to spread message on air pollution as a risk factor		
	Involve health sector in raising awareness		
	Educate teachers	Educate educators	Strategies for raising awareness by frontline workers and educational institutions
	Educate social workers		
	Auxiliary nurse and midwives (ANMs) as messengers	Messaging by frontline workers	
	ASHAs to raise awareness		
	Engage schools in small pilot projects	School-based awareness initiatives	
	Schools to raise awareness of students		
	Children as a channel to reach families	Children as behavioural change agents	Strategies for behavioural change
	Children are best ambassadors		
	Focus on behavioural change in rural areas	Behaviour change communication	
	Panchayat to reinforce behavioural change		
	Empower women to reinforce behavioural change		
	Masks for reducing exposure	Personal protective measures	
	Technological solutions for personal protection such as air filters		
Strategies for research	Develop in-house research	Strengthen research systems	Strategies for building research
	Allocate funds for research for children		
	Health professionals to generate evidence showing health impact	Health professionals as researchers	
	Health professionals to compile evidence systematically		
	Digitize health data at all levels	Data collection and analysis	
	Multicentric collaborative research		
	Cost-benefit studies	Cost-benefit analysis	
	Cost-benefit analysis		
	Conduct health impact estimation studies	Health impact & mitigation	Strategies for knowledge translation
	Research to mitigate ill effects at micro level		
	Establish correlation between air pollution and health impacts		
	Need for solution-oriented research than problem-oriented research	Interventions and solutions	
	Research on solutions/interventions		
	Research on impact of air pollution	Measuring Impact on children	
	Research in air pollution and paediatric health		
	Research on how to protect children		
Strategies for capacity building	Train teachers to educate children	Train educators	Strategies for capacity building of frontline stakeholders
	Train teachers to educate families		
	Build capacity of professors and teachers		
	Train peripheral health workers	Train grassroots workers	
	Train ASHA workers		
	Train medical practitioners; capacity building of paediatricians	Train health professionals	Strategies for capacity building of health sector
	Increased availability of diagnostics tests across hospitals	Strengthen health facilities	
	Increase number of beds in hospitals		
Strategies for policymaking, implementation, and enforcement	School closure not a good policy	School closure policies	Policymaking for schools
	School closure not a solution		
	Provide alternatives for school closure	Alternative policies to school closure	
	Need for alternative policy instead of school closure		
	Need for children-specific policies	Child health-centric policies	Dual strategies for children and health in policy making
	Lack of specific policies targeting children		
	Health to be part of the National Clean Air Program (NCAP)	Integrate health in policymaking	
	Health-centric approach in all developmental policies		
	Integrate health policy with pollution mitigation measures		
	Provide subsidies to farmers for Parali (agricultural stubble) disposal	Government subsidies for clean fuel	Policy making for financial support
	Provide subsidies for cleaner fuel		
	Reduce poverty to access clean fuel	Social and economic initiatives	
	Subsidies for low socioeconomic strata		
	Improved access to healthcare for lower socio-economic strata		
	Finances for climate change & air pollution	Financial resources for climate change and air pollution mitigation	
	Invest in climate mitigation to co-benefit air pollution		
	Policies on number of vehicles sold	Sector-specific policies	Sectoral policy making
	Addressing sectoral policies		
	Advocating climate change as a health issue	Strategies for health, climate change & air pollution	
	Make climate change part of health sector response		
	Stringent laws and punishments for non-conformers	Government based enforcement	Strategies for enforcement and implementation
	Stringent enforcement of laws		
	Enforcement needs public participation	Community-based enforcement	
	Community as a watchdog and regulatory authority		
	Policies to have plan for implementation	Implement policies	
	Executing bodies like panchayat to implement		
	Community to push government for sustainable development	Advocacy by community	Advocacy in policy making
	Policy needs to be driven by public		
	Healthcare professionals in advocacy	Advocacy by health sector	
	Health sector in policy-making		
	Make alternatives accessible	Providing sustainable alternatives	Comprehensive policy making
	Make alternatives available		
	Provide affordable alternative options		
	Providing sustainable alternatives to biomass		
	Well-planned policy rather than knee-jerk reactions	Long-term planning	
	Plan long-term interventions/strategy		
	Need for multiple interventions together	Multipronged strategies	
	Focus holistically		
Strategies for coordination and collaboration	Learn from international experience	International collaboration	Strategies for vertical collaboration
	Consider global obligations and inputs		
	Integration between doctors, schools and ministries	Stakeholder collaboration	
	Involve all stakeholders at national, state and district levels		
	Enhance communication between stakeholders		
	Coordination between centre and state government	Centre-state collaboration	
	Local government to adopt central government schemes		
	Intersectoral convergence	Intersectoral collaboration	Strategies for horizontal collaboration
	Intersectoral integration		
	Interstate collaboration	Domestic regional collaboration	
	Need for air pollution committee for Indo Gangetic Plain (IGP) states		
	Need for inter-ministerial task force	Interministerial coordination	
	Need for grassroots coordination between ministries		
	Policy for collaboration between various ministries		
Strategies for evaluation	Identify hotspots to target resources	Surveillance and hotspot identification	Strategies for monitoring and surveillance
	Conduct surveillance to find hotspots		
	Strengthen monitoring at schools	General monitoring	
	Need for action-oriented monitoring		
	Need better monitoring in rural areas		
	Evaluate policies using tools	Evaluate policies performances	Strategies for evaluating policy based interventions
	Test effectiveness of policies		
	Tools to evaluate impact of interventions at state level	Evaluate interventions	
	Know the results of various interventions		
Strategies for mother and children health	Improve respiratory health of children		Physical health of children
	Vaccination against acute respiratory infection (ARI)		
	Improve Water, Sanitation & Hygiene (WASH)		Environmental health of children
	Improve sanitation		
	Focus on better health of mothers		Build mother's health
	Intervention at life cycle approach		
	Monitor children with underlying respiratory disorders		Children with special needs
	Protect children with underlying disease		
	Counselling sessions for children with respiratory illnesses		

Narrative synthesis of the key findings

Strategies for Emission Reduction

Two significant subcategories extracted under this category were reducing indoor emissions by using clean technology and fuel and ambient air pollution by reducing emissions from various sectors like power, industry, agriculture, waste, construction, transport and health. Participants mentioned the need to switch to FDG technology, use of cleaner fuel and technology and optimising signal systems to protect children’s health from harmful emissions.

“It's really critical to address this challenge and especially for women and children because they are the most impacted, like I said National Green Corps and clean cooking technologies” ~ Participant 10

*“You see all kinds of vehicles emitting large amounts of emissions which you can actually see so the option is for electric vehicles, hybrid vehicles. We can do it, you do car-pooling, you use public transport”* ~ Participant 4

Strategies for Exposure Reduction

Exposure reduction of children in three important microenvironments (schools, home, and transit) was one of the key findings. The participants mentioned the need to reduce travel time, improve ventilation and prevent smoking indoors, more green belts in schools, use smart boards, build schools away from hotspots, and stop the idling of buses to reduce exposure of children.

*“For children, three places are important. Home, schools, and the transit”* ~ Participant 2

*“School should focus on proper ventilation of classrooms”* ~ Participant 3

*“Inside the house, we can use some air filter, preferably equipped with HEPA filter. We should not light up the angeethi or something to prevent smoking inside the house” *~ Participant 15

Strategies for Education and Communication

Public awareness through community, families, and children, effective communication through effective messaging and tools, health sector communication and awareness by and for the health sector, raising awareness of frontline workers through teachers, schools, and grassroots level workers, and behaviour change by means of communication, personal measures, and children were significant findings. Raising awareness through the incorporation of guidelines in the curriculum, participation in eco clubs, and continued medical education could be useful to protect children. Participants mentioned the use of children as messengers and influencers to reach the parents and community at large. Grassroots-level workers can significantly bring about a change in behaviour as they are trusted voices from the same community and socioeconomic background. Effective communication is the way to bring about behavioural change.

*“Children are also found to take back the messages and they influence parents' decisions”* ~ Participant 17

“*So for that, we really need to have a behavioural change intervention and for that, we need community participation. Local self-help groups, panchayat, other local bodies, you know, so that will help to sustain this behaviour of use of clean fuel. Because user clean fuel is not only supply issue, it's a behaviour issue”* ~ Participant 6

Strategies for Research

The subcategories such as “strategies for building research” and “strategies for knowledge translation” focus on developing and strengthening data to find health impacts on children, the role of health professionals as researchers, conducting cost-benefit studies, health impact and mitigation studies, and solution-oriented research.

*“Health people (*health professionals)*, the first is to obviously generate evidence that can impact child health significantly”* ~ Participant 13

*“You got data gaps to it or that we have to fill those gaps. Otherwise, research strategies cannot be made"* ~ Participant 8

*“So, basically I think in India, research is still lacking, first of all associating the pollution levels with the respiratory illnesses. So, we need to have more data in that regard.” *~ Participant 14

Strategies for Capacity Building

Capacity building of frontline stakeholders like educators, grassroots workers, and the health sector through training of health professionals and strengthening the health facilities was found important for protecting children’s health. Training of local health workers like Accredited Social Health Activists (ASHAs), auxiliary nurse midwives (ANMs), and doctors at Primary Health Centers (PHCs) have a deeper reach and can significantly impact the rural population. Training doctors in the field of respiratory medicine, increasing hospital bed capacity, and increasing the availability of diagnostic tests will help in the management of the crisis on high-pollution days.

*“We can have more people who are trained in respiratory medicine in the pediatric population and availability of pulmonary function tests in the hospital set-up.”* ~ Participant 14

*“And like, we also have to look into capacity building of in-service personnel who are working with the health system whether they are medical doctors, specialists, paediatricians, nurses, or community health workers like ASHAs.”* ~ Participant 7

Policy Interventions, Implementation, and Enforcement

This category mentions the need for child-centric policies as there are none at the moment. Also, a holistic policy formulation and long-term planning with health as the main agenda is needed instead of short-term, knee-jerk reactions. It is also important that we evaluate the policies for their effectiveness to fill in the gaps. Also, fiscal subsidies, advocacy by community and health professionals, and enforcement would be required to protect children’s health.

*“It would be important to kind of have maybe some kind of guidelines for even child health.”* ~ Participant 12

*“So, what happens transiently in India is that there is school closure during the pollution months. But I am really not sure whether this would be a good policy.”* ~ Participant 1

*“There are no specific plans, especially for children, child health protection.”* ~Participant 9

Collaboration and Coordination

This category with subcategories such as “vertical and horizontal collaboration” at national, regional and global levels was one of the important findings. Frequent interactions, common action points involving all stakeholders encompassing clinicians, schools, and policymakers, and grassroots coordination between all ministries is needed. In addition, global partnerships and coordination across geographies and sovereign boundaries are also warranted.

*“The NCAP focuses on urban centres as air pollution is mostly thought to be in urban areas. There has to be a specific plan for creating awareness and also addressing pollution from the burning of biomass. So there has to be, I feel, some separate plans for rural areas. That is possible from the Ministry of Rural Development, Environment Ministry, and Health Ministry. These three should come together to have some separate actions for the rural population.”* ~ Participant 9

*“And that health perspective can only come if there's a conversation between health and non-health actors. A classic example is our own NCAP program, the National Clean Air program. It was talking to about eight ministries, but not the health ministry.”* ~ Participant 12

Strategies for Evaluation

This category includes "strategies for monitoring and surveillance” and “strategies for evaluating policy-based interventions” which focus on the need for strengthening monitoring and surveillance systems and evaluating the effectiveness of policies and interventions for addressing the health of children.

“So tools like this which can help evaluate the policies, you know as to how this much effect would be there you know if we are able to reduce it from this level to this level, okay this would be the positive impacts so if these tools are being used by the local policy makers not only at the national level because India is a huge country we need to better go down to the states and the districts.” ~ Participant 11

*“There are so many interventions going on. Right. But we do not have any idea how much they actually produce results.”* ~ Participant 2

Strategies for Maternal and Child Health

This category has been derived through an inductive process and is an innovative category that has been explored in this phase of the research. It focuses on building the health of children through nutrition, vaccination, exercise, and better eating habits. There is also a need to focus on environmental sanitation and maternal health through intervention in a life cycle approach. Also, there should be a special focus on children who have underlying disorders.

*“Then the monitoring of the children who are already suffering from the respiratory health point, they are asthmatics and have other chronic lung disorders. So you monitor them for any exacerbations, treat those children, have adequate kind of alarms or you can teach them how to resuscitate during these months."* ~ Participant 1

*“First we improve the nutrition of the children; if we improve the sanitation and promote hand hygiene (WASH), we then talk about the life cycle approach. The life cycle approach means you start from adolescent girls to women in reproductive age, pregnant women, and newborns. So this is a life cycle approach. You need to intervene in this entire life cycle.”* ~ Participant 6

## Discussion

The strategies extracted from document analysis of the authors' previous study [13] were reemphasized by in-depth interviews based on the objective of the current study. The strategies were for reducing indoor emissions and multisectoral emission reduction, strategies to reduce exposure at home, schools, and transit, strategies for public awareness, effective communication, health sector communication and awareness, and raising awareness by frontline workers and educational institutions, strategies for capacity building of health sector and frontline stakeholders, strategies for building research and knowledge translation, strategies for vertical and horizontal collaboration, strategies for child-centric policies, school closure policies, fiscal policies, comprehensive policymaking, sectoral policymaking, advocacy in policymaking, strategies for monitoring, and strategies for mother and child health.

Our study showed that reducing emissions by means of clean domestic strategies such as adopting clean fuel and clean technologies in addition to reducing exposure could help to protect children’s health. A report by Public Health England 2019 also mentions that policies and interventions targeting air pollutants encompass actions including reducing emissions (prevention) and avoiding individual exposure (avoidance) [12].

Our study highlighted the need for the involvement of grassroots/frontline-level workers. In India, the NCAP was launched in 2019 with the aim to reduce the concentration of PM2.5 and PM10 by 20-30% by 2024 taking 2017 as the base year [17]. A study by Kumari mentions the need to decentralize the said NCAP and strengthen grassroots-level workers and frontline workers for more effective implementation [18]. 

Awareness generation of the community, families, and health professionals was an important factor in the adoption of clean fuel for cooking, better cooking practices, and orchestrating design interventions such as better-ventilated houses. Similarly, awareness generation for farmers in the context of *parali* burning (the annual practice of easy disposal of agricultural waste after crop harvest) and waste management were found critical. A study by Gurjar points to the need for initiating public awareness programs in both rural and urban areas to reduce harmful activities such as open burning of wastes, crop burning, use of biomass as a fuel for cooking, and burning of plastic and rubber materials during winters [19]. A study by Naz et al. found that 56% of children under the age of five years remained with their mothers during cooking, leading to increased exposure and health impacts [20]. They suggested the role of behavioral interventions leading to the use of clean technology and fuels, keeping children away while cooking, and improved ventilation in the house to reduce the impact of household air pollution on children’s health.

As per the results of our study, there is a need to focus on important microenvironments such as homes, schools, and transit to prevent exposure of children. Children spend a significant proportion of their daily time indoors at schools and on daily commutes to and from schools [12]. This concept is also mentioned in a briefing by the European Environment Agency 2023 that preventing exposure of children is the most effective way of protecting their health [12]. Thus, policies should be aimed at improving air quality in child-centric settings and during commutes and sports can reduce their exposure. 

This study showed that proper design of schools, traffic management around schools, and town planning are important elements for preventing exposure of children. A similar study by Prashant et al. found that the proximity of schools to main roads and engine idling by the vehicles at drop-off/pick-up hours create pollution hotspots and increase the exposure of children at schools [21]. Another study by Osborne et al. suggests the use of appropriate interventions at schools to include measures such as zoning, physical distancing from pollution-prone zones, discouraging the use of fossil fuel-based transportation systems from the vicinity of schools, anti-idling campaigns, car-free zones around schools, greening “barrier plants”, creating green belts within school premises, use of walking and bicycles, and scheduling of outdoor learning and play in low traffic hours helps to reduce exposure of school children [22].

Our study mentions the importance of behavioral change communication aimed at the community at large, families, and individuals by creating awareness. Rawat and Kumar also mention that behavioral interventions in the form of citizen science campaigns, environment education programs, etc., amongst relevant stakeholders inter alia help in reducing the exposure of school children [23]. 

Our findings mentioned the need for raising awareness in the community and families, especially children by means of education and using children as behavioral change agents. Kalisa et al. also found the need to raise public awareness in general and children in particular to orchestrate behavioral change [8]. According to them, children need to be sensitized through an appropriate educational curriculum to bring about the said behavioral change [8]. Engaging stakeholders and institutions involved in childcare, children's education, and healthcare could result in the desired behavioral change in children [12]. 

Our findings state that an effective way to reduce HAP was by awareness generation by frontline change agents to adopt cleaner fuels and providing recurring fiscal aid to families to maintain the said shift to cleaner fuels. The Government of India launched the Pradhan Mantri Ujjwala Yojana (PMUY) scheme in 2014 with the objective of moving women away from traditionally used unclean fuels with the provision of free first liquified petroleum gas (LPG) connection to mitigate the adverse health impacts caused by household air pollution [24]. Tripathi et al. found that the main barriers to the use of LPG by poor households were affordability, accessibility, and awareness [25]. Many Ujjwala beneficiaries didn’t adopt LPG as a regular cooking fuel due to the recurring costs of refills [25].

Our study revealed the need for regional cooperation between states, especially Indo-Gangetic Plain (IGP) states. One participant (No.2) mentioned that “*If you look into this figure, every state in this Indo-Gangetic plane, you see almost 40-50% or slightly more, I mean 45-50% contribution is the state's own; remaining is from outside*”, whereby he meant that the IGP states are struggling to find a solution as a large proportion of their air pollution is contributed by neighboring states and interventions in their own state proves ineffective to combat the larger regional issue. This is also corroborated by the participant's own study in which he mentioned that “IGP regions are prone to the transboundary movement of pollutants” [26]. A few others also mentioned the need for interministerial, stakeholder, and sectoral collaboration with the Ministry of Health being the nodal ministry to handle this issue. A similar study by Ganguly et al. mentioned the significance of inter-departmental, inter-city, and cross-state coordination for the management of this issue [27]. It also highlights the need for a single body that could be made accountable for this issue and that there is a need for setting up a regional airshed management authority that enjoys cross-state jurisdiction for the management of this issue and says that there should also be a division of specific roles viz planning, implementation, enforcement, and monitoring amongst various bodies/ministries [27].

Participants in our study mentioned that the health sector should take the lead in policy formulation and health should be central to all policymaking. Greer et al. also mentioned that health as a nodal in policymaking would bring health and non-health actors on board to achieve health goals and would prevent the health sector from struggling to respond to deficiencies arising from other sectors [28].

Boothe et al. mention that in resource-constrained countries in the global south such as India, targeted interventions based on data and evidence-based planning models are essential for achieving optimal results [29].

Recommendations

Based on the findings of this study, the following measures are recommended to protect the health of children from air pollution.

There is a need to frame guidelines for indoor air pollution as the focus of governmental interventions to control pollution has largely been in the realm of outdoor pollution. Programs addressing pollution should be decentralized for them to percolate to the grassroots level. Such a bottom-up approach encompassing union and state government-level policy formulation would result in better district and sub-district level implementation in collaboration with a top-down approach.

Frontline/grassroots-level workers need to be engaged for effective messaging and for better implementation of the programs since they bear the same socio-economic backgrounds as the local populace and use similar narratives and diction thus making communication more effective. Such grassroots workers include those such as ASHA workers, ANMs, Panchayati Raj institution stakeholders, PHC and community health center (CHC) functionaries, and teachers to promote awareness in the community for behavioral change.

There is a need for better collaboration and coordination between various ministries such as ministries of education, urban development, environment forest and climate change, rural development, and health. Each of these ministries needs to appoint a senior-level nodal officer who represents the ministry in standing inter-ministerial meetings. An inter-ministerial task force needs to be made with a steering committee type of formulation which should meet at regular intervals. The health ministry needs to take the lead in all policymaking and policymaking needs to have health in all policy agendas. There is also a need for better dialogue between various ministries for effective policymaking and avoiding duplication of initiatives and wastage of resources. The government needs to have a targeted policy focus under the health ministry as a nodal ministry instead of multiple initiatives under various ministries. Roles and accountability between various stakeholders should be distributed both vertically between various ministries and horizontally between states and urban local bodies. However, the nodal health ministry should take the lead in monitoring the implementation and effectiveness of interventions.

There is also a need to strengthen the data collection and specific surveillance systems for better research which could result in effective evidence-based policymaking.

To combat HAP, the initiative to wean women off traditional polluting fuels such as biomass by LPG cylinders should be continued with an added thrust to make such behavioral shift durable through subsidies for refills, thereby reducing the cost burden on the poor households. Making biogas available by roping in private companies through corporate social responsibility is another modality for such change by means such as the one-village-one-company model.

There is a need to encourage community participation through awareness generation and behavioural change communication which could lead to enforcement of pollution control measures by polluting entities. Existing policies should be evaluated for their effectiveness by identifying policy gaps and thereafter filling those gaps with appropriate policies. There is also a need to address the vulnerable within vulnerable groups so as to include children with underlying respiratory disorders and those from lower socio-economic strata.

Strengths and limitations

To the best of our knowledge, the present study is the first qualitative research to explore the strategies for protecting children’s health against air pollution in India based on the experiences of policymakers, academicians, researchers, and health experts. The current study incorporates the insights of most of the conceivable relevant stakeholders such as policymakers, health professionals, academicians, scientists, non-governmental organization (NGO) members, functionaries in multilateral agencies, and researchers. There were a few policymakers, scientists, and academicians who were familiar with air pollution and health. So, finding appropriate persons who could be included in our research was very difficult. Another limitation was the difficulty in getting appointments for interviews with high-level scientists and researchers. They were often busy and the authors had to approach them in advance for seeking interviews, which was time-consuming.

## Conclusions

The study finds that in order to mitigate pollution-related adverse health impacts, adequate focus needs to be given to environments where children spend most of their time. While most existing strategies have focussed on reducing the sources of pollution, adequate attention has not so far been paid to reducing exposure and building physical health. There is also a need for holistic policy formulation with vertical and horizontal coordination between various stakeholders at global, national, and regional levels. Further, a policy intervention beyond governmental measures is needed to bring about behavioral change by focussing on promoting awareness through educational institutions, the health sector, and grassroots-level workers. There is a need for strengthening research and building capacity at various levels. The government should focus on comprehensive policymaking with a special focus on children’s health and their microenvironments.

It seems that despite having access to cell phones and the internet through computers, the awareness of the multiple online initiatives launched by the government for awareness generation is not adequate. This points to the need for wider communication and resultant penetration by the government in this direction. In addition, customized strategies targeting the rural landscape are needed to enhance the reach of pollution control programs. In any event, rural areas have the additional challenges of poor digital connectivity. Local urban bodies like gram panchayat, ASHA workers, and ANMs could be effective change agents in such targeting. Lastly, the government must offer accessible, available, and affordable alternatives to existing high-polluting causalities.

## Appendices

List of questions

The list of questions that the participants were asked in interviews were:

Q 1. What educational and communication strategies, in your opinion, can help mitigate the adverse health impacts of air pollution on children’s health in India?

Q 2. What emission reduction strategies can help mitigate the adverse health impacts of air pollution on children’s health in India?

Q 3. What exposure reduction strategies can help mitigate the adverse health impacts of air pollution on children’s health in India?

 Q 4. What capacity-building strategies can help mitigate the adverse health impacts of air pollution on children’s health in India?

Q 5. What monitoring and feedback strategies can help mitigate the adverse health impacts of air pollution on children’s health in India?

Q 6. How can policymaking, implementation, and enforcement strategies help to mitigate the adverse health impacts of air pollution on children in India?

Q 7. In your opinion, how can collaboration and coordination help to mitigate the adverse health impacts of air pollution on children in India?

Q 8. What research strategies can help to mitigate the adverse health impacts of air pollution on children in India? and in your opinion, how can research help to mitigate the adverse health impacts of air pollution on children in India?

Q 9.  What else, in your opinion, can help mitigate the adverse health impacts of air pollution on children in India?

## References

[1] Manisalidis I, Stavropoulou E, Stavropoulos A, Bezirtzoglou E (2020). Environmental and health impacts of air pollution: a review. Front Public Health.

[2] (2024). WHO: Household air pollution. https://www.who.int/news-room/fact-sheets/detail/household-air-pollution-and-health.

[3] Kaur R, Pandey P (2021). Air pollution, climate change, and human health in Indian cities: a brief review. Front Sustain Cities.

[4] Prabhakaran P, Jaganathan S, Walia GK (2020). Building capacity for air pollution epidemiology in India. Environ Epidemiol.

[5] (2019). The impact of air pollution on deaths, disease burden, and life expectancy across the states of India: the Global Burden of Disease Study 2017. Lancet Planet Health.

[6] Dutta A, Jinsart W (2022). Air pollution in Delhi, India: it's status and association with respiratory diseases. PLoS One.

[7] Ghorani-Azam A, Riahi-Zanjani B, Balali-Mood M (2016). Effects of air pollution on human health and practical measures for prevention in Iran. J Res Med Sci.

[8] Kalisa E, Clark ML, Ntakirutimana T, Amani M, Volckens J (2023). Exposure to indoor and outdoor air pollution in schools in Africa: current status, knowledge gaps, and a call to action. Heliyon.

[9] (2024). UNICEF. CHILDHOOD AIR POLLUTION EXPOSURE KEY MESSAGES. Childhood Air Pollution Exposure: Key Messages.

[10] Chowdhury S, Chaudhary E, Dey S (2024). Protecting child health from air pollution in India. Indian Pediatr.

[11] (2024). Children in India 2018. Children in India 2018: A Statistical Appraisal.

[12] Agency EE (2024). European Environment Agency: Air pollution and children’s health. https://www.eea.europa.eu/publications/air-pollution-and-childrens-health.

[13] Wahi DC, Sohrabizadeh S, Jahangiri K (2024). Exploring the strategies for mitigating the adverse health impacts of air pollution on community: a document analysis [IN PRESS]. Heliyon.

[14] Elo S, Kyngäs H (2008). The qualitative content analysis process. J Adv Nurs.

[15] Palinkas LA, Horwitz SM, Green CA, Wisdom JP, Duan N, Hoagwood K (2015). Purposeful sampling for qualitative data collection and analysis in mixed method implementation research. Adm Policy Ment Health.

[16] Salehi S, Ardalan A, Ostadtaghizadeh A, Garmaroudi G, Zareiyan A, Rahimiforoushani A (2019). Conceptual definition and framework of climate change and dust storm adaptation: a qualitative study. J Environ Health Sci Eng.

[17] Gopikrishnan G, Kuttippurath J (2024). Four years of National Clean Air Programme (NCAP) in Indian cities: assessment of the impact on surface ozone during the period 2018-2022. Sustain Cities Soc.

[18] Kumari S (2021). National Clean Air Programme, 2019: critical analysis. Nyaayshastra Law Rev.

[19] Gurjar BR (2024). Air Pollution in India: Major issues and challenges. https://www.magzter.com/stories/Education/Energy-Future/Air-Pollution-In-India-Major-Issues-And-Challenges.

[20] Naz S, Page A, Agho KE (2016). Household air pollution and under-five mortality in India (1992-2006). Environ Health.

[21] Kumar P, Omidvarborna H, Barwise Y, Tiwari A (2020). Mitigating Exposure to Traffic Pollution in and Around Schools: Guidance for Children, Schools and Local Communities. Mitigating Exposure to Traffic Pollution in and Around Schools: Guidance for Children, Schools and Local Communities.

[22] Osborne S, Uche O, Mitsakou C, Exley K, Dimitroulopoulou S (2021). Air quality around schools: Part I - a comprehensive literature review across high-income countries. Environ Res.

[23] Rawat N, Kumar P (2023). Interventions for improving indoor and outdoor air quality in and around schools. Sci Total Environ.

[24] Selvam V, Ashok D, Rajamanoharan ID, Rajalakshmi V, Vidhya K (2022). Impact of Ujjwala Yojana scheme and its effect on behavioural changes among rural women. Int J Asian Bus Info Manage.

[25] Tripathi A, Sagar A (2019). Ujjwala, V2.0: what should be done next?. https://static1.squarespace.com/static/59f2a2038a02c746600f6bbb/t/5d0503a7562f010001cc373f/1560609704122/Ujjwala+V2.0+Jun+19b.pdf.

[26] Nair N, Dey S, Bherwani H, Ghosh AK (2022). Long-term changes in aerosol loading over the ‘BIHAR’ State of India using nineteen years (2001-2019) of high-resolution satellite data (1 × 1 km2). Atmos Pollut Res.

[27] Ganguly T, Kurinji L, Guttikunda S (2020). How Robust are Urban India’s Clean Air Plans: An Assessment of 102 Cities. An Assessment of.

[28] Greer SL, Falkenbach M, Siciliani L, McKee M, Wismar M, Figueras J (2022). From health in all policies to health for all policies. Lancet Public Health.

[29] Boothe VL, Baldauf RW (2020). Traffic emission impacts on child health and well-being. Transport and Children's Wellbeing.

